# Using Rural–Urban Continuum Codes (RUCCS) to Examine Alcohol-Related Motor Vehicle Crash Injury and Enforcement in New York State

**DOI:** 10.3390/ijerph16081346

**Published:** 2019-04-15

**Authors:** Joyce C. Pressley, Leah M. Hines, Michael J. Bauer, Shin Ah Oh, Joshua R. Kuhl, Chang Liu, Bin Cheng, Matthew F. Garnett

**Affiliations:** 1 Departments of Epidemiology and Health Policy and Management and Center for Injury Epidemiology and Prevention at Columbia, Columbia University, New York, NY 10032, USA; 2 Bureau of Occupational Health and Injury Prevention, New York State Department of Health, Albany, NY 12237, USA; leah.hines@health.ny.gov (L.M.H.); Michael.bauer@health.ny.gov (M.J.B.); matthew.garnett@health.ny.gov (M.F.G.); 3 Department of Epidemiology, Columbia University, New York, NY 10032, USA; so2461@caa.columbia.edu (S.A.O.); jkuhl650@gmail.com (J.R.K.); nkchangliu@gmail.com (C.L.); 4 Department of Biostatistics, Columbia University, New York, NY 10032, USA; bc2159@cumc.columbia.edu

**Keywords:** rural health, motor vehicle crash, injury, alcohol, traffic citations and enforcement

## Abstract

Rural areas of New York State (NYS) have higher rates of alcohol-related motor vehicle (MV) crash injury than metropolitan areas. While alcohol-related injury has declined across the three geographic regions of NYS, disparities persist with rural areas having smaller declines. Our study aim was to examine factors associated with alcohol-related MV crashes in Upstate and Long Island using multi-sourced county-level data that included the Crash Outcome Data Evaluation System (CODES) with emergency department visits and hospitalizations, traffic citations, demographic, economic, transportation, alcohol outlets, and Rural–Urban Continuum Codes (RUCCS). A cross-sectional study design employed zero-truncated negative binominal regression models to assess relative risks (RR) with 95% confidence interval (CI). Counties (*n* = 57, 56,000 alcohol-related crashes over the 3 year study timeframe) were categorized by mean annual alcohol-related MV injuries per 100,000 population: low (24.7 ± 3.9), medium (33.9 ± 1.7) and high (46.1 ± 8.0) (*p* < 0.0001). In multivariable analyses, alcohol-related MV injury was elevated for non-adjacent, non-metropolitan counties (RR 2.5, 95% CI: 1.6–3.9) with higher citations for impaired driving showing a small, but significant protective effect. Less metropolitan areas had higher alcohol-related MV injury with inconsistent alcohol-related enforcement measures. In summary, higher alcohol-related MV injury rates in non-metropolitan counties demonstrated a dose–response relationship with proximity to a metropolitan area. These findings suggest areas where intervention efforts might be targeted to lower alcohol-related MV injury.

## 1. Introduction

Declines have been reported in alcohol-related motor vehicle (MV) crashes in New York State (NYS) over the last decade; however, improvements have not been uniform across the three distinct NYS geographic areas: New York City (NYC), Long Island, and Upstate (the rest of NYS) [1,2,3]. The counties comprising these regions vary in the degree to which they possess characteristics previously reported to be associated with alcohol-impaired driving, crash risk and crash-related injury and mortality [4,5,6,7,8,9,10,11,12]. 

Counties can vary widely with regard to transportation options available and affordable to those consuming alcohol in on-premises alcohol establishments, as well as in the types of roadways and environmental conditions impaired drivers may encounter. There are noted socioeconomic disparities between metropolitan and non-metropolitan areas that we theorize combine to contribute to the alcohol-related MV crash disparities observed across the three major areas of NY State. We hypothesize that this occurs through multiple interacting factors [4,5,6,7,8,9,11,12]. Rural areas may have fewer resources with which to implement interventions such as sobriety checkpoints and may conduct other visible law enforcement activities less frequently with less intensity than better-resourced geographic areas [4,10]. Similarly, rural or less metropolitan areas may have fewer resources with which to provide affordable public transportation alternatives to driving [6,7]. Sparsely populated areas, such as are found in non-metropolitan areas, are challenging for transit systems to operate acceptable and useful schedules that coincide with after-hours social needs. Extended hours for public transportation in metropolitan areas have been associated with reduced incidence of impaired driving. The largest alcohol-related MV crash improvements have been observed in NYC where incomes are higher and private taxis, livery, ride hailing services, and affordable public transportation alternatives are readily available.

In combination, lower median household incomes that inhibit the growth and availability of taxis and ride-hailing services and poorly-resourced law enforcement in the environment of sparse affordable transportation is problematic. This may contribute to lower identification and ticketing of impaired drivers, lowered use of interlocks and other interventions as well as gaps in monitoring of interlock compliance [2,3,4,6,8]. Cultural norms that lead to lowered restraint use and environmental road conditions, such as two-way traffic corridors that may be curvy, less well-lighted and less traveled can increase both single vehicle crash risk as well as contribute to lower detection and to Emergency Medical Service (EMS) delays once a crash has occurred [4,5,6,7,8]. Emergency medical and trauma services may be at a greater distance with increased travel times and be less equipped to address severe injury and thus contribute to higher mortality in rural areas [11,12].

Smaller historic declines in alcohol-related crashes have been observed in Long Island, with the smallest declines observed in Upstate. Upstate, defined here as NYS counties outside of NYC and Long Island, accounts for 42% of NYS population, has 51% of NYS licensed drivers and 59% of alcohol-impaired driving crashes resulting in injury or death [3,13]. During the timeframe of this study, ride-sharing services, such as Uber and Lyft, were banned from operating in counties outside of NYC, where, for the most part, public transit systems and taxi services are limited [14]. Although ride-hailing services have had exponential growth in other geographic areas across the nation, and despite showing promise for doing so, reports of the impact on alcohol-related MV crashes have not been uniform [15].

The aims of this study are: (1) to identify counties in the Upstate and Long Island regions of NY State with higher rates of alcohol-related MV crash injury; (2) to examine county-level population, transportation, enforcement and economic factors hypothesized to contribute to disparities; and (3) to identify areas where focused intervention efforts might be effective in addressing NYS alcohol-related MV crash injury.

## 2. Materials and Methods

We employed a cross-sectional study design using the NYS Crash Outcome Data Evaluation System (CODES) [13] containing medical and law enforcement information with other multi-sourced county-level characteristics to examine our hypotheses related to factors associated with disparities in alcohol-related MV injury rates observed in regions of NYS. Geographic differences in rurality, socioeconomics, transportation alternatives and law enforcement exist between the regions. Specifically, in the two less urban regions of NYS, Long Island and Upstate, we employ multi-sourced county-level data [16,17,18,19,20,21], including categories created from Rural–Urban Continuum Codes (RUCCs) [22], to examine county-level factors associated with alcohol-related injury across low, medium and high alcohol-related MV injury counties.

### 2.1. Data Sources

NYS CODES data links police and motorist MV crash reports to discharge records from hospital inpatient care and emergency department visits collected through the Statewide Planning and Research Cooperative System (SPARCS). Multiple identifying factors such as gender, date of birth, social security number and name are used to link individuals from the crash reports to the hospital records. Probabilistic linkage then uses the identifying factors to estimate the probability that two of the records are in fact the same person from the same event through use of the software, LinkSolv 8.3 (Strategic Matching, Inc., Morrisonville, NY, USA), a product from Strategic Matching, Inc. LinkSolv, used widely to link records for many different purposes, has been used extensively by traffic safety researchers to prepare and link police crash records, EMS ambulance response records and emergency department and inpatient hospital treatment records [13,23]. Cases require both a crash report as well as an inpatient or outpatient record. Probabilistic linkage uses identifying factors to estimate the probability that two of the records are in fact the same person from the same event. The CODES linkage is robust, with strong and consistent linkage probabilities, and a high sensitivity for accurately linking cases. Based on calculations performed using the Strategic Matching, Inc. (Linksolv 8.3), there was a sensitivity of 99.96% and a specificity of 82.98% pooled across three linkage iterations. 

All county-level injury and mortality data provided by the NYS Department of Health (NYS DOH) [13] was merged with a county-level analytic data set built by aggregating and then merging publicly available data sources [16,17,18,19,20,21]. These data sources contain MV traffic citations, alcohol establishment characteristics, transportation, socioeconomic variables and roadway characteristics using several available data sources for NYS.

The NYS DOH provided county-level total and alcohol-related MV injuries (emergency department visits and hospitalizations) and deaths from the CODES [13]. The NYS Department of Motor Vehicles (NYS DMV) provided data through a public website on citations and licensed drivers that were categorized by citation and aggregated to the county level [16]. NYS DMV data included all citations from 2011–2014 that were issued to motorists for violations of NYS Vehicle and Traffic Law (VTL), Thruway Rules and Regulations, Tax Law, Transportation Law, Parks and Recreations Regulations and NYS Penal Law from 2011 to 2014 [16]. Records of all persons in each county of NYS who hold or were issued driver licenses and permits were also obtained from the NYS DMV [18]. The quarterly list of active liquor licenses for all businesses permitted to sell alcohol for consumption on-site or off-premises in NYS, by county, was obtained from the NYS Liquor Authority [17]. Population density and demographic data at the county level, including age/sex composition of the population and annual household income were obtained from the American Community Survey, U.S. Census Bureau [20]. Roadway characteristics, such as roadway network composition, were extracted from the NYS Highway Mileage Report, produced by the NYS Department of Transportation [21].

### 2.2. Study Population

The study population included NYS residents and persons involved in a motor vehicle crash in one of the 57 counties outside of NYC (population 11,244,669) which comprised the geographic area of the study. The CODES study population with alcohol-related MV injury ranged in age from <1 to 93 years old and without an alcohol-related injury from <1 to 95 years. There was a total of 56,000 alcohol-related MV injuries over the three-year timeframe of the study. 

#### 2.2.1. Variable Definitions

##### Outcome(s)

*Total MV injury rates.* The mean annual rate of injuries from CODES data for MV occupants per 100,000 population was calculated for injuries resulting in death or that were treated in an emergency department or were hospitalized, 2012–2014. 

*Annual alcohol-related MV injury rates.* The mean annual number of alcohol-related MV injuries and deaths per 100,000 persons was calculated using CODES alcohol-impaired traffic crashes from 2012 to 2014. 

*Proportion of alcohol-related injury/total injury.* Calculated at the county level, the proportion of alcohol-related MV occupant injury was the total alcohol-related injuries/deaths divided by total motor vehicle occupant injuries/deaths.

##### County Categorizations

*County categorization by mean annual alcohol-related injury rates.* Counties were classified into three categories based on the annual alcohol-related MV injury rate per 100,000 residents: low (*N* = 16 counties, alcohol-related MV injury rates, mean 24.7 ± 3.9, approximately <30 per 100,000), medium (*N* = 19 counties, alcohol-related injury rates, mean alcohol-related MV injury, 33.9 ± 1.7, approximately 30–37 per 100,000), and high (*N* = 22 counties, mean alcohol-related MV injury, 46.1 ± 8.0, approximately 37–71 per 100,000). Counties in the *Low category* included: Albany, Broome, Chemung, Erie, Essex, Madison, Montgomery, Nassau, Onondaga, Putnam, Rensselaer, Rockland, Schenectady, Tioga, Tompkins and Westchester. Counties for the *Medium category* included: Cattaraugus, Cayuga, Chautauqua, Chenango, Clinton, Columbia, Cortland, Dutchess, Franklin, Niagara, Orange, Oneida, Orleans, Oswego, Saratoga, Schoharie, Suffolk, Ulster and Wayne. Counties in the *High category* included: Allegany, Delaware, Fulton, Genesee, Greene, Hamilton, Herkimer, Jefferson, Lewis, Livingston, Monroe, Ontario, Otsego, St Lawrence, Schuyler, Seneca, Steuben, Sullivan, Warren, Washington, Wyoming and Yates.

*Rural–Urban Continuum Codes (RUCCs).* RUCCs were available at the county level as a nine-category classification scheme with three metropolitan and six non-metropolitan categories [22]. The latest available census data was used to categorize metropolitan counties by population size and non-metropolitan counties by degree of urbanization and adjacency to a metropolitan area. The RUCCs allow counties to be classified into finer groups with the aim of capturing metropolitan influence on less urban areas. We collapsed the nine RUCCs into three categories of counties: (1) metropolitan area; (2) non-metropolitan, adjacent to a metropolitan region; (3) non-metropolitan, non-adjacent to a metropolitan region.

##### Demographics

*Population per square mile.* Population density per square mile was obtained from census data for each county [20].

*Percentage of population by age group.* The proportion of the total county population included those who were aged 0–14 years or who were 70 years and older. 

*Annual household mean and median income, in $10,000.* The estimated household mean and median county-level income for a 12-month period was divided by 10,000 [20].

*Seating position.* Occupant seating position, previously noted to be associated with injury and mortality, was analyzed by individual seating positions for the front and rear seating positions [24,25]. Due to sample size, categories were collapsed into front seated-driver, front seated-passenger and rear-seated passenger. 

##### Transportation Variables

*Taxi drivers per 100 population.* The number of for-hire taxi driver licenses (classes E and EM) was calculated per 100 county residents for each county [21].

*Taxi vehicles licensed per 100 population.* The number of taxi vehicles licensed in a county per 100 county residents was used as a measure of the availability of taxis as an alternative to driving. 

*Taxi vehicles licensed per mile.* The number of taxi vehicles licensed per roadway mile was calculated for each county.

##### Roadway Characteristics

*Percent city road.* Proportion of city road mileage was obtained using total miles from the NYS highway mileage report as the denominator [21].

*Percent county road.* The proportion of county road mileage was obtained by dividing the total mileage of county roads by the total road miles using the publicly available NYS highway mileage report [21].

##### Alcohol Variables and Measures

*Alcohol outlets per 1000 population.* The total number of businesses in each county with active liquor licenses permitting the sale of alcohol per 1000 population, was dichotomized by whether alcohol is sold for on-premises consumption, such as bars and restaurants, or whether it is sold for off-site consumption, such as liquor stores [17]. 

*On-premise alcohol outlets.* The number of businesses per county with active liquor licenses that sell alcohol for on-premises consumption was calculated per 1000 county population and per road mile of public roads [17].

*Off-premise alcohol outlets.* The number of businesses per county with active liquor licenses that sell alcohol only for off-premises consumption was calculated per 1000 county population and per road mile of public roads [17].

##### Enforcement Related Variables and Measures

*Moving citations*. Total moving violations included those issued between 2011 and 2014 (*n* = 14,312,885 citations) at the county level to NYS resident motorists aged 16–95 years. Moving violations included running red lights, stop signs, signals, lane changes, speeding, failure to yield right-of-way, driving while ability-impaired (DWAI), violating interlock mandates and other citations issued for moving violations of traffic laws. Violations relating to registration, inspection, insurance, parking and driver licensure were excluded from moving violations [16].

*Alcohol-related citations*. Citations pertaining to alcohol-involved moving violations included driving while intoxicated, DWAI, ignition interlock-related violations, driving under the combined influence of drugs and alcohol and refusal to submit to a breathalyzer test. These were obtained from the publicly available NYS DMV citation database and were included in our analysis of alcohol-related moving traffic citations. The proportion of alcohol-related citations per moving citation was calculated at the county level [16].

*Interlock enforcement.* Total citations for any of the recorded interlock citations included having someone else breathe into the interlock, driving a vehicle without an interlock, assuming a false identity to avoid interlock, renting a vehicle with the intent of avoiding the interlock, and dismantling, disabling or removing the interlock or otherwise circumventing the driver’s license restriction to drive only an interlock equipped vehicle. The proportion of alcohol ignition interlock-related citations to total citations and to total alcohol-related citations were calculated at the county level [16].

#### 2.2.2. Statistical Analysis

Data sources were merged at the county level for the 57 counties comprising this study. Descriptive statistics were examined for driver, traffic citations/violations, total crashes, and county characteristics across the alcohol-related MV injury categories of low, medium and high. Available covariates were selected for statistical models based on our hypotheses and the literature. Bivariate analyses were examined for variables with 0.20 significance and hypothesized to be of importance. Zero-truncated negative binomial models were used to analyze the alcohol-related MV injury [26,27]. This method was chosen because the outcome of our study, the number of alcohol-related MV injuries, was greater than zero in every jurisdiction. The zero-truncated negative binomial was selected over the zero-truncated Poisson model because the former could handle over dispersion and typically fit the data better. The analysis was conducted using PROC FMM of SAS version 9.4 (SAS Institute, Cary, NC, USA) [27]. A *p*-value <0.05 was considered statistically significant. 

## 3. Results

### 3.1. Study Population

The geographic area examined included more than 11 million NYS residents living in 57 counties. The mean age of the alcohol-related MV injury population (*n* = 56,000 over the 3 year study timeframe) was 35.75 ± 15.42 years and the median age was 33.00 years. The majority of those involved in alcohol-related crashes were males (64.26%). There was no statistically significant difference in the percentage of adolescents, children or older adults across the injury categorized counties, although there was a trend for those aged 70 years or older to have an inverse relationship with alcohol-related MV injury (*p* = 0.06) (Table 1). Household income was inversely associated with alcohol-related MV injury (Table 1) and was significantly higher in metropolitan and areas adjacent to metropolitan areas than in non-adjacent areas (Table 2).

More than 5 million residents were in counties categorized as low alcohol-related MV injury, nearly 4 million resided in the medium category and nearly 2 million resided in the high injury category (Table 1). 

### 3.2. Alcohol-Related Motor Vehicle (MV) Injury

**Figure 1:**
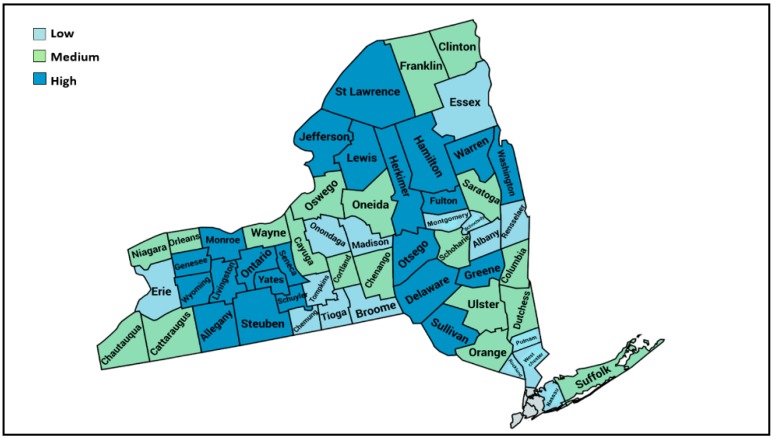
Classification of counties into low (light blue), medium (green) and high (dark blue) annual alcohol-related MV injury rates per 100,000 population.

The three MV injury categories were grouped at the county level by mean alcohol-related MV injury per 100,000 population (Table 1) into low, medium and high alcohol-related MV injury (*p* < 0.0001) (Figure 1). Mortality tended to be approximately three-fold higher in the higher injury categories compared to the low injury category (*p* = 0.06) (Table 1). 

As injury categories increased, the proportion of the counties classified as metropolitan decreased (Table 1). Compared to counties with low alcohol-related MV injury rates, counties with medium and high alcohol-related MV injury categories were significantly more rural as measured by population density per square mile (Table 1). This was consistent with the RUCC categorization as well where nearly 88% of counties in the low alcohol-related MV injury category were classified as metropolitan compared to 58% in the medium and 36% in the high MV alcohol-related injury category.

### 3.3. Alcohol Citations

When examined across low, medium and high injury categories, there was no difference in the percentage of interlock citations to total alcohol-related citations (*p* = 0.79) (Table 1). When examined by RUCC category, increasing distance from a metropolitan area was associated with a significant decrease in the proportion of interlock citations among all alcohol-related citations (*p* = 0.037) (Table 2). 

**Figure 2:**
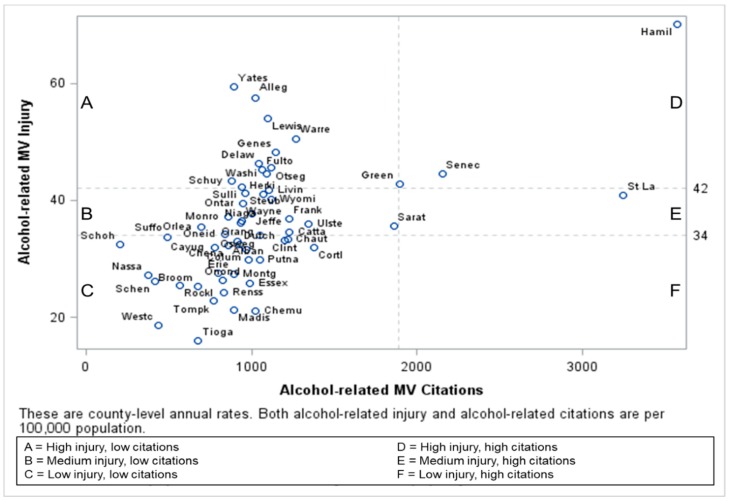
County-level annual rates of alcohol-related MV injury by alcohol-related citations in NYS per 100,000 population.

**Figure 3:**
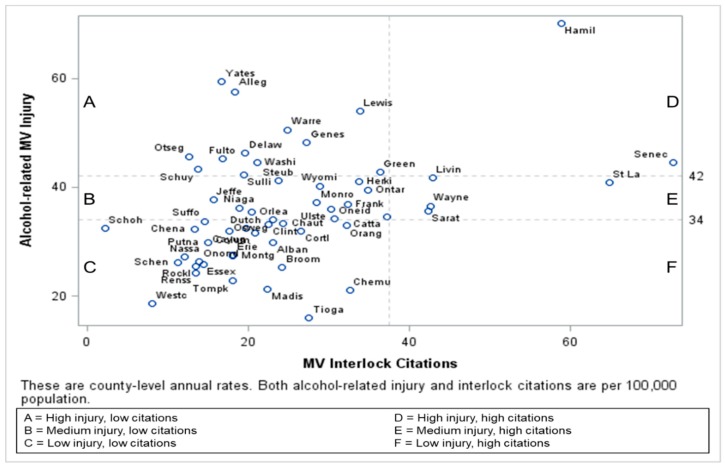
County-level annual rates of alcohol-related MV injury by interlock citations in NYS per 100,000 population.

Although citations given due to an alcohol-related crash event versus at a non-crash vehicle stop could not be differentiated, counties in the higher alcohol-related injury categories issued significantly more alcohol-related citations per 100,000 population (*p* = 0.005) and as a proportion of all moving violations (*p* = 0.02) (Table 1). County-level annual rates of alcohol-related MV injury by alcohol-related violations are shown in Figure 2 and by interlock citations in Figure 3.

Two of the three counties with the highest rates of alcohol-related injury are in the areas not adjacent to a metropolitan area (Figure 1), had similar annual interlock citations per 100,000 population as other RUCCs and had a significantly lower percent of interlock citations among their alcohol-related citations (*p* = 0.037). 

### 3.4. Alcohol Establishments

There were no differences in per capita alcohol premises across low, medium and high alcohol-related MV injury categories (Table 1). However, when examined as premises per road mile, both on-premise and off-premise alcohol establishments showed a significant inverse relationship with alcohol-related MV injury (Table 1). An analogous relationship was observed when examined by RUCC category where there were no per capita differences in on-premise or off-premise alcohol establishments, but a significant relationship between being more metropolitan and having more alcohol-related establishments per road mile (Table 2). 

### 3.5. Roadway Characteristics

There was no difference in the percentage of roads defined as city roads across all alcohol-related MV injury categories (Table 1).

### 3.6. Taxi Licenses

Taxi licenses, whether examined per road mile or per capita, demonstrated a significant inverse relationship with alcohol-related MV injury category (Table 1). The low injury category had twice as many taxis per 100 population as the high injury category and more than seven times more per road mile. There were no ride hailing services (e.g., Uber, Lyft) offering pickup services in the 57 counties during the timeframe of this study. 

When examined by RUCC category, taxi licenses were significantly higher in the more metropolitan areas. Although taxis were slightly more prevalent in areas adjacent to metropolitan areas than in rural areas, the two non-metropolitan areas, adjacent and non-adjacent, had a significantly smaller fraction of the taxis per road mile and were more similar to each other than to the metropolitan RUCC category (Table 2). 

### 3.7. Occupant Seating Position

Front seated occupants accounted for the majority of alcohol-related injuries (87% or higher) in the low, medium, and high MV injury categories (Table 1). When examined across RUCC categories, in the non-adjacent RUCC category, drivers tended to comprise a smaller proportion of occupants (69%) than in the metropolitan or adjacent to a metropolitan region (75%) (Table 2). 

### 3.8. Independent Predictors of Alcohol-Related MV Injury

The zero-truncated negative binomial model of alcohol-related MV injury was adjusted for DWAI citations, taxi licenses, and RUCC categories. In adjusted models, DWAI citations showed a small, but significant inverse relationship with alcohol-related MV injury after controlling for RUCC and taxi licenses. The relative risk in non-metropolitan, non-adjacent areas was significantly elevated at 2.5 times higher than in metropolitan areas (Table 3) while non-metropolitan areas adjacent to a metropolitan area tended to have a higher, but insignificant, relative risk (56%) for alcohol-related MV injury than metropolitan counties.

## 4. Discussion

This study used multi-sourced data to identify counties in the Upstate and Long Island regions of NYS with high rates of alcohol-related MV crash injury. Higher alcohol-related MV injury rates occurred in non-metropolitan counties and demonstrated a dose-response relationship with proximity to a metropolitan area. In addition to inter-regional disparities, this study documents significant intra-regional differences and provides clues to areas where focused intervention efforts might be effective in lowering NYS alcohol-related MV injury. Increasing distance from a metropolitan area was associated with more alcohol-related citations per 100,000 population, but there was no difference across regions in the proportion of alcohol-related citations to total moving citations and there was a significantly lower proportion of interlock citations to total alcohol-related citations in the less metropolitan areas. Although numbers were small, neither on-premise or off-premise serving alcohol establishments per 100,000 population differed across the RUCC categories, but there were significantly fewer transportation alternatives—fewer licensed taxis—in the less metropolitan areas. 

A unique feature of the study is that it used linked hospital and police data to identify and categorize counties into three levels of alcohol-related MV injury and then examined how population, transportation, enforcement and economic factors differed across the three levels of alcohol-related MV injury. Earlier work had demonstrated disparities and an uneven decline in alcohol-related MV injury across the three major regions comprising NYS (NYC, Upstate, and Long Island) [1,2,3]. This study extends those findings and documents that disparities also exist within the sub-regions comprising the large number of counties in Upstate where metropolitan areas and areas adjacent to metropolitan areas experience lower alcohol-related MV injury than nonadjacent areas.

An inverse association was observed between alcohol-related MV injury and proximity to a metropolitan area. In the Long Island region, metropolitan areas exhibited the lowest alcohol-related MV injury rates, followed by non-metropolitan areas adjacent to a metropolitan area, with the highest alcohol-related MV injury rates being in non-metropolitan areas not adjacent to a metropolitan area. This pattern was observed despite metropolitan areas having a significantly greater number of alcohol establishments per road mile. Although this finding is consistent with previous examinations of the three major areas comprising NYS that show metropolitan NYC to have the lowest rates of alcohol-related MV injury [1,2,3], this extends the findings of previous reports which did not examine the large Upstate region’s 55 counties by RUCCs, socioeconomic, transportation or population characteristics.

Our study demonstrated an inconsistent association with alcohol establishment selling density depending on whether it was measured per capita or per road mile. Per capita serving for on-premise consumption and selling for off-premise consumption showed a non-significant bivariate relationship to MV injury, while alcohol-related MV crash injury per road mile showed an inverse relationship for both on-premise and off-premise alcohol establishments. Previous studies in California and elsewhere found positive associations between bar density and risk of alcohol-related MV crashes [28,29,30,31,32] although it should be noted that some of these studies also included property damage only crashes as well as those producing injury or did not control for other environmental factors such as rurality, geographic proximity to an urban area, socioeconomic factors or alternative transportation availability and accessibility. 

The finding of lower availability of alternative transportation such as taxis and lower income levels, suggests that the impact of recently introduced ride hailing services, not available during the timeframe of this study [15], may be muted by lower income levels and affordability. Alternative transportation may be limited in rural areas contributing to lower use of taxi services in counties with fewer licensed taxicabs if the wait time is prohibitively long or costs are prohibitively high.

The availability and affordability of alternative forms of transportation, such as subways, buses, ride hailing services, safe ride services and taxis, have been noted previously to impact DWAI rates [32]. Expansion of the hours of operation of the Washington, DC Metro by three hours was associated with a decrease in the probability of being arrested for a DWAI in neighborhoods with at least one bar within 100 meters of a metro station. The number of alcohol consumers who chose to drive home decreased by nearly 20%. Other results from a small study analyzing surveys collected from users of safe ride services at bars found that safe ride services attract patrons at high risk for DWAI [32]. These reports, coupled with the findings of our study that show increased alcohol-related MV injury in areas with limited transportation alternatives, suggest that offering alternative forms of transportation may have a significant role in reducing impaired driving.

We observed higher alcohol-related citations in counties in the medium and high alcohol-related injury categories compared to counties with low alcohol-related injury, but we were not able to differentiate those given because of a crash. Similarly, alcohol-related citations comprised a higher proportion of all moving traffic citations in the medium and high alcohol-related injury categories. Since the implementation of the Child Passenger Protection Act of 2010 (Leandra’s Law), any person convicted of driving while intoxicated (DWI)/DWAI in NYS is mandated to have an alcohol ignition interlock device installed on all vehicles they own or operate [33]. While alcohol citations were higher in geographic areas with increased injury, there was no difference observed in the proportion of interlock citations among alcohol-related citations in these areas. In fact, when examined by RUCC category, the proportion of interlock citations to total alcohol-related citations was inversely related to both alcohol-related injury and to metropolitan status. It is not clear whether law enforcement in rural areas, with the highest alcohol-related MV injury, is not detecting, not ticketing or whether rural residents are more compliant with their interlocks.

The implementation of mandatory interlock devices for alcohol driving violations has been reported to lower recidivism while they are in place [34,35,36,37,38,39,40,41]. Drivers enrolled in interlock programs have been reported to be 15%–65% less likely to be re-arrested for driving under the influence (DWAI) [34]. Meta-analysis studies found that alcohol ignition interlock devices are effective at reducing drunk driving recidivism while installed in participants’ vehicles [34,39]. In a randomized trial, a 65% reduction in re-arrests for DWAI was observed in the interlock group compared to a control group [34]. However, other studies have suggested interlocks are effective only as long as they are installed in the vehicles of impaired drivers [35]. A case-control study observed a significant difference in the rate of DWAI recidivism comparing first-time offenders with installed interlocks to first-time offenders without interlocks. The differences in recidivism disappeared when the group with interlocks had them uninstalled [39]. These findings suggest the need for further investigation of this approach as a potential avenue through which less metropolitan areas of NYS might lower their alcohol-related MV injury rates.

### Study Limitations

This study has limitations. We did not stratify by citations issued during a road stop that was not associated with a crash. Future analyses should use the NYS CODES to differentiate citations given in road stops from those issued following a crash to disentangle and differentiate preventive alcohol-related driving enforcement from DWAI citations conferred post-crash due to an alcohol-related crash. We used alcohol-related and interlock-related law enforcement citations. These citations could have been based on breathalyzer, blood or an officer’s sobriety assessment. A major limitation of county-level analyses is that the sample size is small and counties can be heterogeneous in terms of environmental factors and overlook individual neighborhood characteristics. These findings suggest that future analyses with individual level crash data and smaller geographic area detail could be used to address this heterogeneity. Some of the differences in rates near cut points that categorized counties into low, medium and high were small. Another potential limitation is a couple of counties whose nearest hospital is in an adjacent state could contribute to an underestimate of the alcohol-related injury in these counties. However, our analyses that examined and reported proportions of alcohol-related citations to total citations and similarly alcohol-related MV injury compared to total MV injury should not be affected significantly. This study may have missed alcohol-related crashes where injury was minor and did not require medical treatment at an emergency department or hospital or where that medical treatment occurred at an out-of-state hospital. In addition, alcohol-related driving captures only those drivers who were “caught” and may underestimate the true incidence of alcohol-impaired driving.

## 5. Conclusions

Collectively, these findings suggest the need for further study regarding potentially modifiable factors associated with rural alcohol-related MV injury as well as areas where shifting transportation trends could widen existing alcohol-related MV injury disparities. This study identified higher alcohol-related MV injury risk in less metropolitan areas where travel distances can be longer, alternative transportation choices fewer, and costs for those higher. Of importance, although this study was conducted using data prior to the introduction of ride-hailing services in Long Island and Upstate NY, it suggests that future studies should strive to monitor the possibility that socioeconomic disparities observed in this study could lead to a widening of current disparities in alcohol-related injury in poor rural areas.

## Figures and Tables

**Table 1:** County characteristics by alcohol-related motor vehicle (MV) injury category.

Variables	Alcohol-Related MV Injury Category ^1^			*p*-Value
	Low	Medium	High	
Number of counties, *n*	16	19	22	
**Demographics**				
Total population, n	5,352,876	3,898,729	1,993,064	
Percentage of population ages 14 or younger, mean (SD)	17.28 (2.31)	17.19 (1.49)	16.62 (2.00)	0.51
Percentage of population ages 70 or older, mean (SD)	10.72 (1.48)	10.75 (1.36)	11.76 (1.75)	0.06
Population density (per square mile), mean (SD)	829.75 (1203.1)	243.58 (359.97)	121.53 (228.70)	0.007
Rural–Urban Continuum Code (RUCC) category, *n* (%)				0.03
Metropolitan	14 (87.50)	11 (57.89)	8 (36.36)	
Adjacent	2 (12.50)	6 (31.58)	12 (54.55)	
Non-adjacent	0 (0.00)	2 (10.53)	2 (9.09)	
Annual household mean income in $10,000, mean (SD)	8.36 (2.48)	6.85 (1.48)	6.28 (0.51)	0.0008
Annual household median income, $10,000, median (SD)	6.38 (1.78)	5.40 (1.18)	4.98 (0.35)	0.003
**Alcohol-related injury**				
Annual alcohol-related MV injury per 100,000 population, mean (SD)	24.68 (3.86)	33.94 (1.69)	46.05 (7.99)	<0.0001
Annual alcohol-related MV death per 100,000 population, mean (SD)	0.72 (0.80)	1.98 (1.68)	2.37 (2.93)	0.06
Annual MV death rate per 100,000 population, mean (SD)	7.79 (9.08)	13.06 (10.74)	49.97 (121.88)	0.17
**Seating Position**				
Front seat—Driver	3016 (72.66)	3025 (76.53)	1846 (73.69)	0.0053
Front seat—Passenger	637 (15.35)	560 (13.94)	404 (16.13)	
Rear Seat—all positions	462 (11.14)	383 (9.53)	255 (10.18)	
**Alcohol-related citations**				
Annual alcohol-related citations per 100,000 population, mean (SD)	762.96 (219.52)	1003.66 (357.04)	1341.17 (737.88)	0.0045
Percentage of alcohol-related citations among moving citations, mean (SD)	4.13 (1.11)	5.20 (1.26)	5.31 (1.50)	0.021
Annual interlock citations per population, mean (SD)	17.87 (6.55)	24.85 (10.17)	30.25 (16.57)	0.01
Percentage of interlock citations among alcohol-related citations, mean (SD)	2.42 (0.78)	2.47 (0.81)	2.31 (0.77)	0.79
**Alcohol outlets**				
On-premise alcohol outlets per 1000 population, mean (SD)	1.65 (0.69)	1.79 (1.05)	2.15 (1.09)	0.28
On-premise alcohol outlets per road mile, mean (SD)	0.21 (0.13)	0.12 (0.06)	0.09 (0.05)	0.0003
Off-premise alcohol outlets per 1000 population, mean (SD)	1.33 (1.16)	1.22 (1.01)	1.19 (0.70)	0.88
Off-premise alcohol outlets per road mile, mean (SD)	0.18 (0.19)	0.08 (0.05)	0.05 (0.03)	0.002
**Transportation**				
Percentage city road, mean (SD)	64.63 (10.91)	62.95 (7.67)	61.41 (8.56)	0.56
Percentage county road, mean (SD)	20.06 (8.41)	20.05 (5.87)	20.27 (4.85)	0.99
Taxi licenses per 100 population, mean (SD)	1.37 (1.22)	0.80 (0.61)	0.65 (0.57)	0.027
Taxi licenses per road mile, mean (SD)	2.70 (3.88)	0.80 (1.21)	0.36 (0.30)	0.004

^1^ Counties were classified using CODES (Crash Outcome Data Evaluation System) data into three categories based on the annual alcohol-related MV injury rate per 100,000 residents: low (*N* = 16 counties, mean 24.68 ± 3.86), medium (*N* = 19 counties, mean alcohol-related MV injury, 33.94 ± 1.69), and high (*N* = 22 counties, mean alcohol-related MV injury, 46.05 ± 7.990).

**Table 2:** County characteristics by Rural–Urban Continuum Codes (RUCCs).

Variables	RUCC Category ^1^			*p*-Value
	Metro	Adjacent	Non-Adjacent	
Number of counties, *n*	33	20	4	
**Population**				
Total population, *n*	9,837,487	1,164,400	242,782	
Population density (per square mile), mean (SD)	571.37 (913.16)	75.10 (38.01)	55.13 (20.27)	0.04
Percentage of population ages 14 or younger, mean (SD)	17.51 (1.93)	16.43 (1.78)	15.60 (1.44)	0.04
Percentage of population ages 70 or older, mean (SD)	10.51 (1.28)	12.24 (1.61)	10.75 (1.17)	0.0003
Annual household mean income in $10,000, mean (SD)	8.36 (2.48)	6.85 (1.48)	6.28 (0.51)	0.003
Annual household median income $10,000, median (SD)	5.98 (1.51)	4.89 (0.40)	4.73 (0.31)	0.004
**Alcohol-related injury**				
Annual alcohol-related MV injury per 100,000 population, mean (SD)	32.50 (9.09)	40.37 (10.19)	43.25 (10.87)	0.007
**Seating position**				
Front seat—Driver	6725 (74.81)	904 (74.77)	213 (69.38)	0.16
Front seat—Passenger	1329 (14.78)	192 (15.88)	56 (18.24)	
Rear seat—Passenger	936 (10.42)	113 (9.48)	38 (12.38)	
**Alcohol-related citations**				
Annual alcohol-related citations per 100,000, mean (SD)	872.05 (310.64)	1371.44 (775.12)	1144.11 (91.89)	0.005
Percentage of alcohol-related citations among moving citations, mean (SD)	4.96 (1.28)	4.73 (1.59)	5.89 (1.16)	0.33
Annual interlock citations per 100,000, mean (SD)	22.69 (10.06)	29.44 (17.21)	21.48 (8.30)	0.17
Percentage of interlock citations among alcohol-related citations, mean (SD)	2.61 (0.85)	2.15 (0.54)	1.86 (0.61)	0.037
**Alcohol outlets**				
On-premise alcohol outlets per 1000 population, mean (SD)	1.74 (0.92)	2.17 (1.13)	1.71 (0.48)	0.28
On-premise alcohol outlets per road mile, mean (SD)	0.17 (0.11)	0.07 (0.03)	0.06 (0.02)	0.0002
Off-premise alcohol outlets per 1000 population, mean (SD)	1.24 (1.09)	1.24 (0.74)	1.20 (0.16)	0.996
Off-premise alcohol outlets per road mile, mean (SD)	0.13 (0.15)	0.04 (0.02)	0.04 (0.02)	0.015
**Transportation**				
Percentage city road, mean (SD)	64.63 (10.91)	62.95 (7.67)	61.41 (8.55)	0.09
Percentage county road, mean (SD)	19.36 (6.84)	21.30 (5.66)	20.75 (2.63)	0.55
Taxi licenses per 100 population, mean (SD)	1.08 (1.00)	0.71 (0.60)	0.47 (0.18)	0.18
Taxi licenses per road mile, mean (SD)	1.79 (2.95)	0.27 (0.23)	0.17 (0.09)	0.049

^1^ Counties were classified using nine Rural–Urban Continuum Codes (RUCCs) into three categories: (1) metropolitan area; (2) non-metropolitan, adjacent to a metropolitan region; (3) non-metropolitan, non-adjacent to a metropolitan region.

**Table 3:** Unadjusted and adjusted relative risk (RR) estimates for alcohol-related injury using a zero-truncated negative binomial model.

Variables	Unadjusted, Alcohol-Related Injury	Adjusted, Alcohol-Related Injury
Interlock citations	0.665 (0.617, 0.716)	-
Driving while ability-impaired (DWAI) citations	0.987 (0.986, 0.989)	0.991 (0.988, 0.993)
Taxi licenses	0.395 (0.307, 0.509)	0.828 (0.629, 1.091)
RUCC Category ^1^		
Metro	Ref.	Ref.
Adjacent	2.286 (0.767, 6.812)	1.563 (0.741, 3.297)
Non-adjacent	4.316 (2.380, 7.828)	2.497 (1.608, 3.878)

^1^ Counties were classified using nine Rural–Urban Continuum Codes (RUCCs) into three categories: (1) metropolitan area; (2) non-metropolitan, adjacent to a metropolitan region; (3) non-metropolitan, non-adjacent to a metropolitan region.
